# Mitochondrial genome in *Hypsizygus marmoreus* and its evolution in Dikarya

**DOI:** 10.1186/s12864-019-6133-z

**Published:** 2019-10-22

**Authors:** Gang Wang, Jingxian Lin, Yang Shi, Xiaoguang Chang, Yuanyuan Wang, Lin Guo, Wenhui Wang, Meijie Dou, Youjin Deng, Ray Ming, Jisen Zhang

**Affiliations:** 1 Center for Genomics and Biotechnology, Haixia Institute of Science and Technology, Fujian Provincial Key Laboratory of Haixia Applied Plant Systems Biology, College of Life Sciences, Fujian Agriculture and Forestry University, Fuzhou, 350002 China; 2 College of Life Sciences, Fujian Agriculture and Forestry University, Fuzhou, 350002 China; 3 College of Crop Science, Fujian Agriculture and Forestry University, Fuzhou, 350002 China; 4 Department of Plant Biology, University of Illinois at Urbana-Champaign, Urbana, IL 61801 USA

**Keywords:** *Hypsizygus marmoreus*, *Basidiomycota*, Mitochondrial genome, SNP, Dikarya

## Abstract

**Background:**

*Hypsizygus marmoreus*, a high value commercialized edible mushroom is widely cultivated in East Asia, and has become one of the most popular edible mushrooms because of its rich nutritional and medicinal value. Mitochondria are vital organelles, and play various essential roles in eukaryotic cells.

**Results:**

In this study, we provide the *Hypsizygus marmoreus* mitochondrial (mt) genome assembly: the circular sequence is 102,752 bp in size and contains 15 putative protein-coding genes, 2 ribosomal RNAs subunits and 28 tRNAs. We compared the mt genomes of the 27 fungal species in the *Pezizomycotina* and *Basidiomycotina* subphyla, with the results revealing that *H. marmoreus* is a sister to *Tricholoma matsutake* and the phylogenetic distribution of this fungus based on the mt genome. Phylogenetic analysis shows that *Ascomycetes* mitochondria started to diverge earlier than that of *Basidiomycetes* and supported the robustness of the hyper metric tree. The fungal sequences are highly polymorphic and gene order varies significantly in the dikarya data set, suggesting a correlation between the gene order and divergence time in the fungi mt genome. To detect the mt genome variations in *H. marmoreus*, we analyzed the mtDNA sequences of 48 strains. The phylogeny and variation sited type statistics of *H. marmoreus* provide clear-cut evidence for the existence of four well-defined cultivations isolated lineages, suggesting female ancestor origin of *H. marmoreus.* Furthermore, variations on two loci were further identified to be molecular markers for distinguishing the subgroup containing 32 strains of other strains. Fifteen conserved protein-coding genes of mtDNAs were analyzed, with fourteen revealed to be under purifying selection in the examined fungal species, suggesting the rapid evolution was caused by positive selection of this gene.

**Conclusions:**

Our studies have provided new reference mt genomes and comparisons between species and intraspecies with other strains, and provided future perspectives for assessing diversity and origin of *H. marmoreus*.

## Introduction

Mitochondria (mt) are vital organelles, and play various essential roles in eukaryotic cells. Since the discovery of mt DNA in 1963, large-scale studies have been performed to analyze their structures and functions. The evolution of the mt genome is fundamentally different from the major groups of eukaryotes (animals, plants, protists and fungi) [1]. Plant mt genomes have high recombination frequencies, including large intergenic regions, introns and their associated intronic open reading frames (ORFs) [2], and the repetitive genomic elements, which therefore causes variations in the mitochondrial (mt) genome size [3] as seen for example, in the *Silene* genus. In contrast, animal mt genomes tend to have higher rates of DNA sequence evolution than in plants [4], and in general their mt genomes are gene rich and have fewer introns [5, 6]. Historically compared to animals and plants, fungal mt genomes have been studied less. With the emergence of sequencing technology in recent years, a continuously increasing number of fungal mt genomes is analyzed, and about 186 complete fungal mt genomes are available at NCBI as of July 2019. This provides a powerful resource for comparative studies to reveal the patterns and mechanisms of mt genome evolution [7].

The fungal mt genome provides important clues to fungal evolution, population genetics and biology because it displays remarkable variations in terms of gene order, genome size, composition of intergenic regions, presence of repeats, introns, and associated ORFs [1, 8]. Previous studies revealed that the genome size of mitochondria ranged from 19 kb to 235 kb [9, 10], which mainly result from the presence or absence of large intronic and intergenic sequences [11] with the number and length of introns being the most predominant factors [12]. One hypothesis suggested that introns were abundant in the ancestral mt genes, and were subsequently lost in most lineages [13]. The mt genomes of conspecific fungi species may also be divergent as indicated through the analysis of mt genomes of 11 *Cordyceps militaris* strains, the sizes of these mt genomes varied from 26.5 to 33.9 kb and the number of introns in genes ranged from two to eight [14]. Mt molecular markers have been successfully applied for evolutionary biology and systematics because mt genomes often evolve faster than nuclear genomes and allow for robust phylogenetic analyses based on the conserved proteins of the oxidative phosphorylation system [15].

In this study, a commercial strain of *H. marmoreus* was sequenced using PacBio sequencing, and a complete mt genome of 102,752 bp was generated. The aims at this study are to (i) present a complete and annotated mt genome sequence of *H. marmoreus*, (ii) compare the mt genome of *H. marmoreus* with the genomes of other fungi in *Pezizomycotina* and *Basidiomycotina* to identify the common and specific characteristics of the mt genomes, (iii) provide insights into the evolution and phylogenetic relationships between different strains of *H. marmoreus* through the analysis of the variations at specific loci.

## Results

### Sequence and general features of the *H. marmoreus* mt genome

**Fig. 1:**
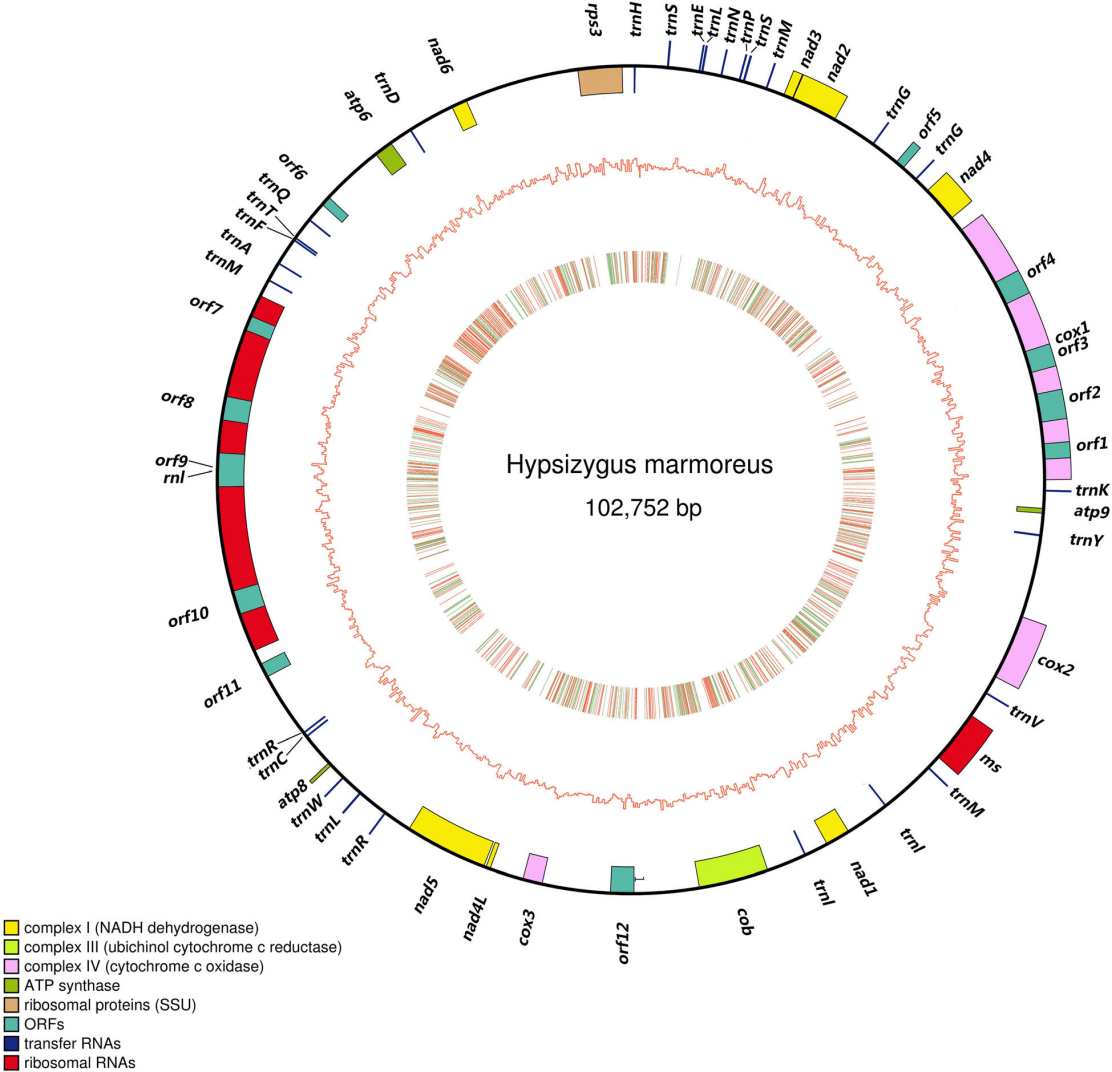
Circular map showing the genomic features of the *H. marmoreus* mt genome. The outer ring shows the gene positions, and the bar outside/inside the ring line indicates if the gene was on the forward/reverse strand of genome sequence. The middle ring shows the GC content of the genome sequence in the histogram. The inner circle shows variant sites in a density map

Based on sequence homology with *Pleurotus ostreatus* mt genome (GenBank:NC_009905) we identified a scaffold with the length of 102,752 bp as mt genome. The complete mt genome of *H. marmoreus* is a circular DNA molecule with a GC content of 32%, containing 56 annotated genes (Table 1, Fig. 1). Of the 56 genes, fifteen genes are conserved protein-coding genes, with known functions in the electron transport chain and oxidative respiration. Seven NADH dehydrogenases (*nad1, nad2, nad3, nad4, nad4L, nad5, nad6*) are subunits of complex I [16]; cob is one subunit of coenzyme Q-cytochrome c reductase, which plays a critical role in the biochemical generation of ATP [17]; three cytochrome c oxidases (complex IV: *cox1, cox2, cox3*) are the last enzymes in the respiratory electron transport chain [18]; three ATP synthase subunits (*atp6, atp8, atp9*) [11], and a ribosomal protein S3 gene, *rps3* which is known to play critical roles in ribosome biogenesis and DNA repair. In addition, twenty-nine non-coding genes, including 27 tRNA and the small and large ribosomal RNA subunits (rns, rnl), were identified. Twelve free-standing ORFs were divided into two categories, eight ORFs in intronic sequences and 4 ORFs in intergenic regions.

**Table 1:** Summary of 56 genes of the *H. marmoreus* mt genome

Gene category	Gene family	Genes number	Gene names
Conserved gene	NADH dehydrogenase subunit	7	nad1,nad2,nad3,nad4,nad4L,nad5,nad6
	Cytochrome c oxidase subunit	3	cox1, cox2, cox3
	ATP synthase subunit	3	atp6, atp8, atp9
	Apo cytochrome b	1	Cob
	Ribosomal protein S3	1	rps3
Predicted gene	ORF in intronic region	8	orf1,orf2,orf3,orf4, orf7, orf8,orf9, orf10
	ORF in intergenic region	4	orf5,orf6,orf11,orf12
Non-coding	Ribosomal RNA	2	rns, rnl
Gene	Transfer RNA	27	tRNA-Gly_01, tRNA-Gly_02, tRNA-Met_03, tRNA-Ser_04, tRNA-Pro_05, tRNA-Asn_06, tRNA-Leu_07, tRNA-Glu_08, tRNA-Ser_09, tRNA-His_10, tRNA-Asp_11, tRNA-Gln_12, tRNA-Thr_13, tRNA-Phe_14, tRNA-Ala_15, tRNA-Met_16, tRNA-Arg_17, tRNA-Cys_18, tRNA-Trp_19, tRNA-Leu_20, tRNA-Arg_21, tRNA-Ile_22, tRNA-Ile_23, tRNA-Met_24, tRNA-Val_25, tRNA-Tyr_26, tRNA-Lys_27
Total		56

**Fig. 2:**
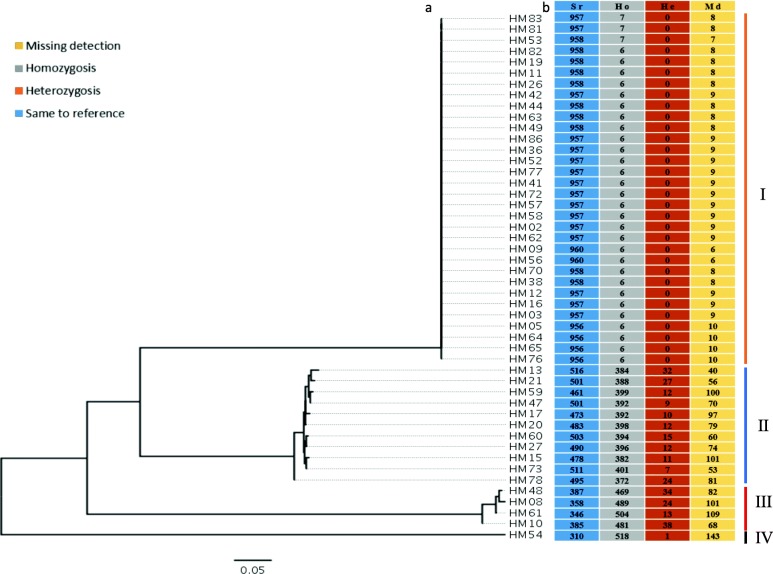
The phylogenetic tree and genotypes of SNPs among 48 *H. marmoreus* strains. **a** Phylogenetic tree of 48 strains using 972 mitochondrial genome SNP sites; **b** Genotypes of 972 mt SNP loci in 48 strains. Blue: homozygous locus which was same as the reference; Gray: homozygous locus which was different to the reference; Orange: heterozygous locus; Yellow: not detected

The 48 *H. marmoreus* dikaryotic strains were sequenced with the Illumina Hiseq2500 platform, and the short reads were aligned to the reference genome to identify variants that were isolated by both GATK and SAMtools [19]. Through the selection of the overlaps from the two methods and a hard filtering step with stringent thresholds, 1373 reliable variant sites including 972 SNPs and 401 InDels, were identified in the mt genome sequence (Fig. 1, Additional file 9: Table S3). According to the SNPs pattern of 48 strains on the SNP loci (Fig. 2, Additional file 10: Table S4, Additional file 11: Table S5), 6964 genotype sites of 48 *H. marmoreus* strains were detected to be homozygous and different to the reference genome. While only 281 sites were detected to be heterozygous, a high ratio of up to 96.12% of the genotypes was homozygous, indicating that the mt genome of *H. marmoreus* existing in dikaryotic cells was a haplotype. A phylogenic tree was constructed based on the 972 mt SNPs from 48 strains with the bootstrap value of 1000 times (Fig. 2, Additional file 3: Figure S3). The phylogenetic tree clearly showed the existence of four clusters that diverged from each other. According to the classification of the cultivated species on the tree, the *H. marmoreus* was divided into three groups (group I, II, III, IV). Group I contained 32 strains with the low bootstrap values, indicating that the mt genomes of these strains are highly conserved, whereas, strain HM54, is an exception, and was separated from all other strains.

Moreover, we performed PCR amplification of mt DNA based on two InDels (Additional file 13: Table S7): one (GGGGTCCCGTAC/G) located at position 93,344 on the mt genome and another (TAGTAA/T) located at position 93,608 of the mt genome, could be used as molecular markers to differentiate the group I from the other groups, verifying the reliability of the mt DNA for the classification of *H. marmoreus* stains.

### Annotation of variants and neutral evolution of the mt genome

Through analysis using snpEFF software, 1942 effects were detected to be caused by 1373 variants (Table 2). Most of the variants (83%) were located in the intergenic region (including the upstream and downstream regions of the gene); of the 17% remaining, 10 and 7% are located in the exon region and intron of the gene, respectively. Among the variants in codons of protein-coding genes, 18 non-synonymous (dN) mutation sites and 50 synonymous (dS) mutation sites were observed. The ratio of non-synonymous to synonymous mutations (dN/dS) was 0.36, much less than 1, indicating that, the general, mt homologous genes were in general undergoing purification selection during the evolutionary process. Of note, genes *atp6, cob, cox1, nad2, nad4L* and *nad5* have a synonymous mutations number > = 3 (Additional file 12: Table S6). Interestingly, the dN/dS ratio of the *rps3* gene has a value of 1.4 (7/5), suggesting rapid evolution was caused by the positive selection of this gene, which may explain the phenomenon that the *rps3* gene was not found in part of other fungal species, such as, *P. ostreatus, T. cingulata* [1]. Frameshift mutations can cause overall changes in the protein sequences and are a type of mutation that has the greatest impact on gene function [20]. Five high impact variant sites were predicted by the SnpEFF software, including three frameshift variants in *rps3*, one frameshift variant in *nad4L* and the loss of 1 stop codon in *nad5* (Table 3, Additional file 12: Table S6). Three InDels on *rps3* were distributed closely (26,970 to 26,987 bp) and were predicted to be high-risk variants. Two genes, *rps3*, and *nad4L*, were selected to verify the variants based on two stains, HM10 and HM54. In *rps3,* a continuous point mutation (ACCCC/TTGCG), a 9 bp deletion (TTTGGGGAG), and an InDel/SNP (C/ATAGC,) were detected based on three pairs of PCR primers (Additional file 13: Table S7). In *nad4L*, a single nucleotide InDel (C/CA) was detected (C/CA) (Additional file 6: Figure S6). These results were consistent with the resequencing analysis results, confirming the variants among the mt DNA genomes and thus supporting the conclusion that these genes were under purifying selection.

**Table 2:** Statistics for the effects of variants

Region of variants	Effect of variants	Impact level	Count	Percent
Exon	Frameshift variant	High	4	0.21%
	Stop lost	High	1	0.05%
	Conservative inframe deletion	Moderate	1	0.05%
	Conservative inframe insertion	Moderate	1	0.05%
	Missense variant	Moderate	18	0.93%
	Synonymous variant	Low	50	2.58%
	Non coding transcript exon variant	Modifier	116	5.98%
Intron	Intron variant	Modifier	139	7.16%
Intergenic	Intergenic region	Modifier	1189	61.26%
	500 bp upstream of gene	Modifier	210	10.82%
	500 bp downstream of gene	Modifier	212	10.92%
Total	–	–	1941	100.00%

**Table 3:** Five predicted high impact variants among *H. marmoreus* strains

Gene	Location	Reference	Variation	Strain and Genotype
rps3	26970	CCCCCA	C	HM13[1/1]^A^,HM15[1/1],HM17[1/1],HM20[1/1],HM21[1/1],HM27[1/1],HM47[1/1],HM54[1/1],HM59[1/1],HM60[1/1],HM73[1/1],HM78[1/1]
rps3	26981	TTGGG	T	HM13[1/1],HM15[1/1],HM17[1/1],HM20[1/1],HM21[1/1],HM27[1/1],HM47[1/1],HM54[1/1],HM59[1/1],HM60[1/1],HM73[1/1],HM78[1/1]
rps3	26987	A	ATAGC,C	HM08[0/1]^B^,HM10[1/1],HM48[1/1],HM61[1/1]
nad5	67784	A	T	HM08[1/1],HM10[1/1],HM48[1/1],HM54[1/1],HM61[1/1]
nad4L	71212	C	CA	HM02[1/1],HM03[1/1],HM05[1/1],HM08[1/1],HM09[1/1],HM10[1/1],HM11[1/1],HM12[1/1],HM13[1/1],HM15[1/1],HM16[1/1],HM17[1/1],HM19[1/1],HM20[1/1],HM21[1/1],HM26[1/1],HM27[1/1],HM36[1/1],HM38[1/1],HM41[1/1],HM42[1/1],HM44[1/1],HM47[1/1],HM48[1/1],HM49[1/1],HM52[1/1],HM53[1/1],HM54[1/1],HM56[1/1],HM57[1/1],HM58[1/1],HM59[1/1],HM60[1/1],HM61[1/1],HM63[1/1],HM64[1/1],HM65[1/1],HM70[1/1],HM72[1/1],HM73[1/1],HM76[1/1],HM77[1/1],HM78[1/1],HM81[1/1],HM82[1/1],HM83[1/1]

Note: A: homozygous; B: heterozygous

### Comparisons with other fungal mt genomes

To study the evolution of mt in major fungal groups, 15 conserved mt protein-coding genes (*atp6, atp8, atp9, cox1, cox2, cox3, nad1, nad2, nad3, nad4, nad4L, nad5, nad6* and *cob*) were collected from 26 representative fungal species from the NCBI database (Additional file 8: Table S2) for phylogenetic analysis. A phylogenetic tree was constructed using the maximum likelihood method (Additional file 1: Figure S1). All nodes in the tree have bootstrap values of 100%, indicating the robustness of the computed tree. The topology of the tree was consistent with the phylogenetic tree constructed using homologous genes with single copies from the whole genome of these species (15 of 27 fungi have been published on NCBI) (Additional file 2: Figure S2), which was supported by the classic taxonomic classification of these fungal species [21].

**Fig. 3:**
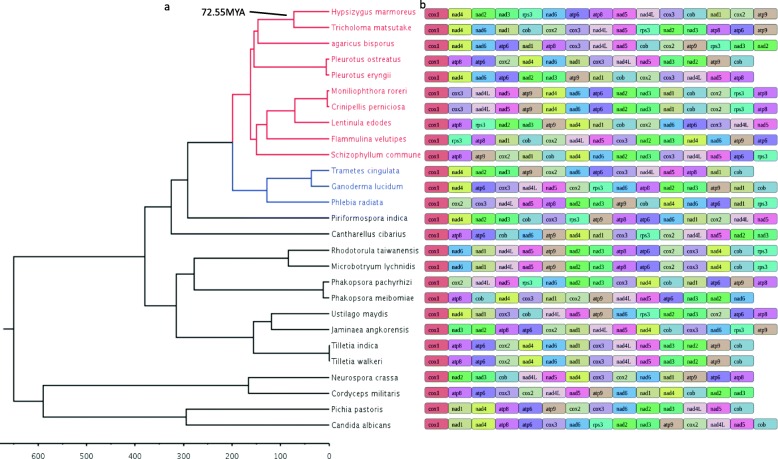
Ultrametric tree of molecular clock analysis and gene order of 27 fungal mt genomes. **a**. The species tree was generated from a concatenated alignment of 14 single-copy orthologous genes (*atp6, atp8, atp9, cox1, cox2, cox3, nad1, nad2, nad3, nad4, nad4L, nad5, nad6* and *cob*). **b**. On the right side of each taxon name is a series of colored boxes representing the mt gene order according to GenBank annotation

Molecular clock analysis based on two fossil calibration points (Fig. 3) and the phylogenetic tree shows that *Ascomycetes* mitochondria started to diverge earlier than that of *Basidiomycetes*. *Agaricomycotina*, *Pucciniomycotina*, and *Ustilaginomycotina* all had similar divergent evolution in *Basidiomycota* (Additional file 1: Figure S1); in *Agaricomycotina*, the genesis of *Agaricales* was later than *Aphyllophorales*. *H. marmoreus* and *T. matsutake* were estimated to have diverged 72.55 MYA, and genetically were the closest among the 27 species. The comparison of gene order shows that the collinearity level of the 15 conserved mt genes did not correlate with the phylogenetic distribution of the 27 fungal species and high gene order variability (Fig. 3). For example, collinearity between *P. pastoris* and *C. albicans* were observed to be higher than that of *T. cingulata* and *G. lucidum*. Also, the *rps3* gene was absent in the mt genome of different phylogenetic groups including *P. ostreatus, P. eryngii, T. cingulate, P. eibomiae, T. indica, T. walkeri, N. crassa, C. militaris*, and *P. pastoris*, suggesting that *rps3* was less conserved in the mt genome of fungi. Consistent with a previous study [1], two sets of genes *nad4L,nad5*, and *nad2,nad3* were found in tandem in all species. These two sets of genes were further observed to be physically located together with 1 bp overlap between the two genes (Additional file 14: Table S8).

## Discussion

Compared to nuclear DNA, mt DNA is much more susceptible to damage and mutations [22]. Mt genomes are very useful genomic resources in evolutionary biology and systematic studies [23, 24] because they evolve faster than nuclear genomes [11, 25], and thus mt genome sequences could be evidence for species determination and classification. In this study, we assembled the first complete mt genome of *H. marmoreus* based on the PacBio sequencing technology. Similar to the mitochondrion of other fungi, the 102,752 bp mtDNA of *H. marmoreus* contained 15 protein-coding genes, 2 rRNAs, 27 tRNAs and 12 ORFs in intrinsic sequences and intergenic regions. In comparison to the 26 fungi species, the gene content of the *H. marmoreus* mt genome is similar, but the gene order exhibits only a limited synteny (Fig. 3), suggesting that extensive genome rearrangements in fungal mt genomes took place. Twenty-seven tRNAs existed in the Mt genomes of *H. marmoreus* and are thought to contribute to mt genome rearrangements as the distribution of tRNAs can change location (Perseke et al. 2008) and associate with breakpoints involved in nuclear chromosomal rearrangements in fungi (Di Rienzi et al. 2009). These 56 genes in the mitochondria of *H. marmoreus* are divided into four clusters (Fig. 1), in which the genes were consistently distributed with either the sense or negative sense strand. This phenomenon also existed in the other examined fungal mitochondria (Additional file 5: Figure S5), suggesting that the phenomenon originated before the origin of fungal species. GC content is an important indicator of genome characteristics, which is related to genetic characteristics and has an important influence on the stability of double-stranded DNA. Invertebrate nuclear genomes, the GC content is significantly positively correlated with genome size [26]. However, in this study, the genome sizes varied from 30 kb to 156 kb, but GC content and genome size showed no significant positive correlation in the 27 examined fungal mitochondria, indicating that the genome sizes were independent of GC content in fungal mitochondria.

In this study, we resequenced the mt genomes of 48 *H. marmoreus* strains. The conspecific phylogenetic analysis revealed that the 48 *H. marmoreus* strains might have originated from three ancestors (Fig. 2). The majority of strains were distributed in group I, suggesting that the diversity of the mt genome in *H. marmoreus* is low. The *H. marmoreus* strains from the wild are few and the majority of them are from artificial domestication and hybridization [27, 28]. The mt genomes provide the evidence for the origin of the female parent. In this study, the 48 *H. marmoreus* stains were phylogenetically distributed in four clusters with HM54 as the out-group, and these four clusters showed different levels of divergence. Probably, the *H. marmoreus* mt genomes were artificially selected from wild species (HM54), and the divergence rate varied among the four clusters of *H. marmoreus.* Moreover, according to the classification of the cultivated species on the tree, the *H. marmoreus* was divided into three groups (group I, II, III) and HM54, group I, which can be distinguished from the group II and group III strains by polyacrylamide gel electrophoresis (PAGE) experiments (Additional file 4: Figure S4). This phenomenon is more common in edible fungi cultivars of *H. marmoreus*, so the variety can be identified by the molecular markers.

Previous studies have suggested that repeat sequences in the intergenic regions have the strongest correlation with gene order; the distribution of tRNAs contributes to protein-coding gene order variation among fungi as they can change location, with the “tandem-duplication-random-loss” being the model for explaining gene order changes [5, 29, 30]. Here, we have shown that there is high variability in terms of how the 15 protein-coding genes are ordered in the mt genome among *Basidiomycetes* and *Ascomycetes*, particularly in basidiomycetes, suggesting a complex interplay of opposing evolutionary forces (Fig. 3). The differentiation of gene order related to divergence time, and the effect of multicollinearity among interspecific species were obvious in the model within a similar differentiation period, but there are also some exceptions, for example, *P. osteratus/P. eryngii, P. taiwanensi/P. meibomiae*, and these results were consistent with the previous report [8].

In this study, similar to *C. militaris* [14], *C. albicans* [31] and *L. kluyveri* [32], the *H. marmoreus* nucleotide variability at intergenic regions (~ 83% of the whole mt genome) was higher than intronic and exon genic regions (~ 17% of the whole mt genome) (Table 2), supporting the hypothesis that most of the variants present in protein-coding genes would be removed by selection [32].

The *rps3* gene was reported to be a common feature of the mt genome [33]. It encodes a ribosomal protein, a component of the 40S subunit and plays a critical role in the initiation of protein translation [34]. Besides, *rps3* had extra-ribosomal activities such as DNA repair, cell signaling, apoptosis/survival and transcriptional regulation [7, 35]. In the 27 fungi species, the *rps3* gene was short of the mt genome of *P. ostreatus, P. eryngii, T. cingulate, P. eibomiae, T. indica, T. walkeri, N. crassa, C. militaris* and *P. pastoris* (Fig. 3). In the bioinformatics analysis of variations, three high frameshift variants were found on the *rps3* gene and the lowly conserved *rps3* was susceptible to high-risk variations (Table 3). The proportion of non-synonymous and synonymous mutations in the *rps3* gene was 1.4(dN/dS > 1), which indicates that the gene sequence was positively selected for and that the gene recently evolved, this being of great significance to species evolution [36, 37]. We also verified one insertion (A/ATAGC, C) using PCR product sequencing in HM10 generating the same result. In the HM54 strain, PCR product sequencing also verified that a 9 bp deletion (TTTGGGAG) and multi-point continuous mutation (ACCCC/TTGCG) occurred between positions 26,975 and 26,989 of the mt genome (Additional file 6: Figure S6). The results suggest a display of unique evolutionary characteristics in *H. marmoreus* as *rps3* is under positive selection. The study argues that it was in part due to elevated rates of evolution in rRNA genes, protein-coding genes were commonly used for the phylogenetic analyses of fungi [1, 11]. As found in the previous study, when cellular ROS levels increase, the mitochondrial genes are highly vulnerable to DNA damage. Increased ROS induces rps3 accumulation in the mitochondria for DNA repair while significantly decreasing cellular protein synthesis. For the entrance into the mitochondria, the accumulation of rps3 was regulated by interactions with HSP90, HSP70, and TOM70 [38]. The specific role of the *rps3* gene in DNA damage repair in *H. marmoreus* and its mechanism requires further study.

Mitochondria, one of the organelles of eukaryotic cells, have their genome and can complete replication, transcription, and translation. In this study, 14 protein-coding genes from the 27 fungi species were used for ML analysis. Combining the ML and Phylogenetic distribution (Fig. 3, Additional file 1: Figure S1, Additional file 8: Table S2) three phylogenetic tree branches according to *Basidiomycetes* (two branches) and *Ascomycetes* sub-kingdom are well-supported, which is in agreement with a previous study [39] that found that *Agaricomycotina* contains 4 orders: *Agaricales*, *Aphyllophorales*, *Sebacinales* and *Cantharellales*. The previous study was based on a single laccase gene and showed that *H.marmoreus* and *F. velutipes* were the closest sisters in *Agaricales* [40]. However, data on single-genes may result in conflicting gene trees and is thus insufficient to reconstruct consistent and accurate phylogenetic hypotheses. Our study based on 14 conserved genes from the mt genome for the phylogeny of 27 fungi species suggested that *H. marmoreus* was the sister to *T. matsutake* which were estimated to have diverged 72.55 MYA, with the multiple gene scale data solving potential issues with gene trees caused by limited gene sets and therefore providing a more accurate phylogenetic classification [41]. The mt genome does not recombine and has the characteristics of matrilineal inheritance. Cluster analysis of *Ciliophora* was conducted using mitochondrial and nuclear genome information, and it was found that the clustering of species was not significantly different, but the tree branches were partially different [42]. Here, the single-copy homologous genes of available 15 fungal genomes published on NCBI taken from the 27 fungi were used to construct the phylogenetic trees and the phylogenetic tree constructed by *Basidiomycetes* and *Ascomycetes* in previous studies [43], which were found to be consistent with the phylogenetic tree of mitochondria (Additional file 2: Figure S2), indicating that the mt genome could be used for the analysis of the evolutionary development of fungi.

In this study, based on phylogenetic distribution and the time tree estimation, *Ascomycetes* mitochondria started to diverge earlier than that of *Basidiomycetes*. A previous study based on six genes estimated that the ancestors of *Basidiomycota* and *Entorrhizomycota* split about 530 MYA [44]. *Agaricomycotina*, *Pucciniomycotina*, and *Ustilaginomycotina* all had similar divergent evolutionary time in *Basidiomycota* which was consistent with previous studies (Additional file 1: Figure S1) [7]. However, our estimation of divergence time was performed on a few calibration points due to the availability of very limited fossil evidence, as the reason, we did not emphasize the divergence time in the *Dikarya*. Further analysis based on calibration points with more fossil evidence would be essential for the prediction of divergence time in *Dikarya* in the future.

## Conclusions

In this study, we provided the first *H. marmoreus* reference sequence of the mitochondrial (mt) genome with a circular structure. Comparative analysis of the mt genomes from 27 fungal species in the dikarya revealed that *H. marmoreus* was a sister to *T. matsutake and Ascomycetes* mitochondria started to diverge earlier than that of *Basidiomycetes*. The correlation between the gene order and divergence time in the fungi mt genomes were observed among the fungal species. Fourteen of fifteen conserved protein-coding genes of mtDNA were revealed to be under purifying selection in the examined fungal species, suggesting the rapid evolution was caused by positive selection of this gene. Moreover, the phylogeny and variation sited type statistics based 48 *H. marmoreus*s trains provide clear-cut evidence for the existence of four well-defined cultivations isolated lineages, suggesting female ancestor origin of *H. marmoreus*. Our study provides the foundation work for assessing diversity and origin of *H. marmoreus* and its evolution in Dikarya.

## Methods

### Fungal strains and DNA preparation

A total of 48 *H. marmoreus* dikaryotic strains (Additional file 7: Table S1) were collected from scientific research institutes, universities, and enterprises. The monocytic strain FQX_MS01 was cultured from a spore of strain HM62. All of these strains were cultured in potato dextrose broth at 25 °C and dark condition for 10-15 days. The mycelia were harvested and washed with sterile deionized water three times, and stored at − 80 °C before processing for DNA extraction. Finally, freeze-dried mycelia were ground with liquid nitrogen and whole genomic DNA extraction was performed using the CTAB method as previously described by Manicon et al. [45].

### Sequencing and assembling of mt genome

We sequenced the whole genome of *H. marmoreus* strain FQX_MS01 using the single-molecule sequencing platform Pacbio RSII (Genomics and Biotechnology Research Center, Fujian Agriculture and Forestry University, Fuzhou, China), producing ~100x Pacbio raw data. Canu [46] was then used for de novo genome assembly, obtaining 51 contigs with a total size of 43,691,898 bp and N50 = 1,760,684 bp. Mt contig was picked out from assembly results through the comparison with mt DNA of *Pleurotus ostreatus* using BLASTX. Some small InDels mistakes were corrected by mapping short reads from Illumina sequencing on raw genome. The mt contig was assembled into a circle molecule based on the overlap of contig ends.

### Mitochondrial genome annotation

The Mfannot web tool (http://megasun.bch.umontreal.ca/RNAweasel/) was used for the annotation of the mt genome. Meanwhile, cDNA sequences collected from RNA-Seq were aligned to the mt genome by PASA [47], and mt homologous proteins were aligned to the mt genome by Genewise. The tRNA genes were identified by tRNAScan-SE [48], and the rRNA genes were identified by the Rfam [49] database. The mt genomic annotation results were manually corrected using the Jbrowse [50] genome browser and WebApollo [51]. OGDRAW [52] and Circos [53] were used to draw the circular map of mt genome.

### Comparative genomics and phylogenetic analysis

The 26 mt assemblies were downloaded from the NCBI database (https://www.ncbi.nlm.nih.gov/genome/organelle/) (Additional file 8: Table S2). Fourteen fully conserved mt proteins, including 4 cytochrome c-oxidases subunits (*cox1, cox2, cox*3 and *cob*), three ATP synthase subunits (*atp6, atp8* and *atp9*) and 7 NADH dehydrogenase subunits (*nad1, nad2, nad3, nad4, nad4L, nad5* and *nad6*) from *H. marmoreus* and the 26 fungal species were used for comparative analysis (Additional file 8: Table S2). Firstly, the homologous protein sequences of all the 27 species were aligned using MAFFT software [54]. Each homologous protein had one alignment result, and all 15 alignments were integrated by joining all sequences into one line for each species. Secondly, the conserved blocks of the alignment were extracted by GBlocks [55]. Thirdly, the best model of protein evolution was determined by ProtTest [56] software. Finally, Maximum likelihood topology searches were completed with RAxML8.1.24 [57] using the model “PROTGAMMALGX”, and analysis was conducted with 1000 bootstrap replicates. Two fossil calibration points were fixed in the molecule clock analysis: the most recent common ancestor (MRCA) of *Agaricus bisporus* and *Schizophyllum commune* diverged 162 MYA [38]; the MRCA of *Candida albicans* and *Cordyceps militaris diverged* 590 MYA [58]. The divergence time of other nodes was calculated by r8s v1.80 [59] software with TN algorithm, PL method and the smoothing parameter value set to 1000 through cross-validation.

### Analysis of strain-specific variants

Each of the 48 dikaryotic strains was sequenced with the Illumina Hiseq2500 platform from an Illumina paired-end library with an insert size of ~ 450 bp, producing 150 bp short reads of up to ~ 3 Gb of raw data. The complete raw data for the genome resequencing of the 48 strains were preprocessed by Trimmomatic [60] and aligned to the mtgenome sequence of *H. marmoreus* strain FQX_MS01 by Bowtie2 [61]. GATKv3.6 and Samtoolsv1.3.1 [62] were both used for SNP and InDel detection with default parameters separately, and the mt variants were selected for this research. Finally, the intersection of the two methods was picked out for a downstream hard filtering with GATK thresholds: QUAL > = 60, QD > = 10.0, MQ > = 13.0, FS < = 20.0, MQRankSum > 3.0 and ReadPosRanSum > = − 3.0, resulting in 972 reliable SNPs and 401 InDels for the next annotation by SnpEff [63]. The alignment of 48 sequences each with lengths of 972 bp was extracted from the 972 SNP sites, and inputted into RAxML v8.1.24 with the parameter “-m GTRCAT” for the construction of a conspecific phylogenetic tree, and bootstrap analysis was conducted with 1000 times resampling. Primer3 software was used to design primers based on mutation site information (Additional file 13: Table S7), with PAGE and Sanger sequencing being used for validation.

## Supplementary information

**Additional file 1: Figure S1.** Phylogenetic analysis of *H. marmoreus* and the other 26 fungal species based on protein sequences of 14 conserved mitochondrial orthologous genes. A max likely hood species tree of 27 fungal species was constructed using RAxML and a bootstrap analysis with 1000 replications was performed. All of the bootstrap values at any node were 100%. The order, sub-kingdom, and kingdom corresponding to each species show their taxonomic classifications.

**Additional file 2: Figure S2.** Phylogenetic analysis of *H. marmoreus* and 14 other fungal species based on a single copy of a homologous gene. A maximum likely hood species tree of 15 fungal species was constructed using RAxML and a bootstrap analysis with 1000 replications was performed. All of the bootstrap values at any node were 100%.

**Additional file 3: Figure S3.** The phylogenetic and SNP analyze of *H. marmoreus* intraspecific. a. Phylogenetic tree construction of 48 HM strains using 972 mitochondrial genome SNP sites; b. Genotypes of 972 mitochondrial SNP loci in 48 HM strains, calculated as diploid. Yellow: the loci are homozygous and consistent with the reference genome; blue: loci are homozygous and inconsistent with the reference genome; red: loci are heterozygous; green: DNA sequencing reads are not aligned at this locus result.

**Additional file 4: Figure S4.** PAGE diagram of PCR products for two InDdels markers in different *H. marmoreus* strains. (A) InDel (GGGGTCCCGTAC/G) located at position 93,608 on the mt genomes; (B) InDel (TAGTAA/T) located at position 93,344 on themt genomes. The strains HM44, HM70 and HM86 were the same as HM62 (references genome) in group I, while, strains HM13, HM8, HM48, HM10 and HM54 were classified in group II, and III. M = marker.

**Additional file 5: Figure S5.** Circle diagrams representing the mitochondrial genomes of 4 different strains.

**Additional file 6: Figure S6.** PCR product sequencing of the variant sites between HM62 and HM10 strains. (A) At 26987 location on the mt genome, there is a 4 bp InDels in *rps3* of HM10. Rps3-2-HM10 and rps3-HM10 are two primers of PCR products. (B) At position 26,975 on the mt genome there is a 4 bp continuous point mutation (ACCCC/TTCGC) and 9 bp deletion (TTTGGGGAG) in *rps3* of HM54. rps3-HM54 and Rps3-2-HM54 are two primers of PCR products. (C) At position 71,212 on the mt genome, there is a 2 bp InDels (C/CA) in *nad4L* of HM10. Nad4L-10 and Nad4L-2-10 are two primers of PCR products.

**Additional file 7: Table S1.** Source of the 48 *H. marmoreus* strains.

**Additional file 8: Table S2.** Statistics for the general features of mt genomes among 26 fungal species.

**Additional file 9: Table S3.** Variation site statistics for the mt genome of *H. marmoreus.*

**Additional file 10: Table S4.** Statistics for the genotypes of 48 strains.

**Additional file 11: Table S5.** SNPs and genotypes of 48 *H. marmoreus* strains.

**Additional file 12: Table S6.** Effects of variants on 15 mt conserved genes.

**Additional file 13: Table S7.** Primers used for *H. marmoreus* DNA amplification and sequencing.

**Additional file 14: Table S8.** The distances between *nad2*, *nad3* and between *nad4L*, *nad5.*

## Data Availability

The mt genome sequences of *H. marmoreus* have been deposited at GeneBank under the accession number of: HM382825 (https://www.ncbi.nlm.nih.gov/nuccore/MH382825.1/).The original genome data was uploaded to NCBI BioProject, under the accession number: PRJNA508399.

## Abbreviations

ATP: Adenosine triphosphate
bp: Base Pair
dN: Non-synonymous
dS: Synonymous
GATK: Genome Analysis ToolKit
MAFFT: Multiple Alignment using Fast Fourier Transform
MRCA: Most recent common ancestor
MYA: Millions of years ago
NADH: Nicotinamide adenine dinucleotide
ORF: Open Reading Frame
PAGE: Polyacrylamide gel electrophoresis
Rfam: RNA families
*rps3*: Ribosomal Protein S3
SNPs: Single nucleotide polymorphism
