# Supplementary figures and images for: Mitochondrial genome in Hypsizygus marmoreus and its evolution in Dikarya
Source: BMC Genomics. 2019 Oct 22;20:765. doi: 10.1186/s12864-019-6133-z (PMC6805638; doi:10.1186/s12864-019-6133-z)

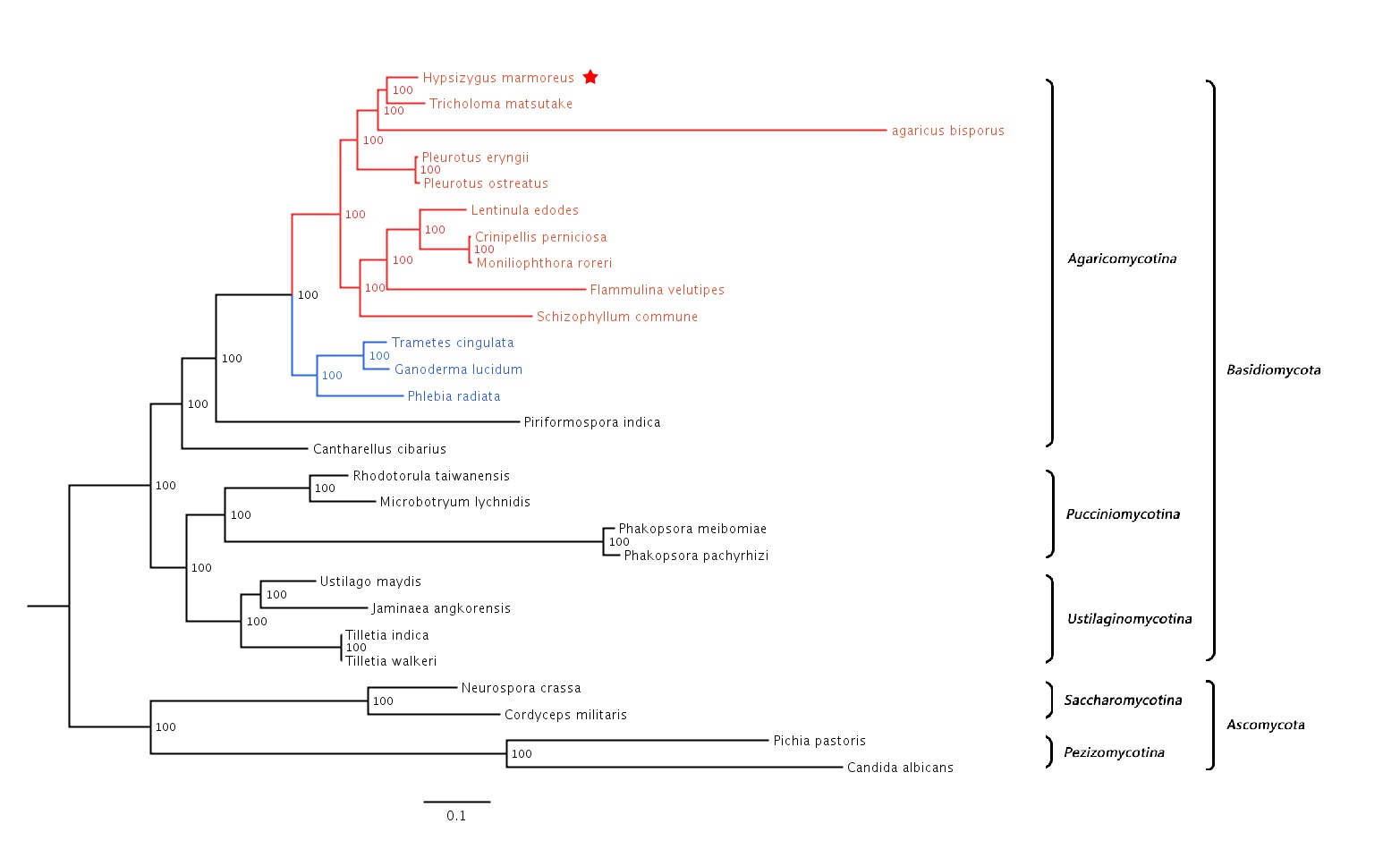

Supplement: Supplementary file 1 — Additional file 1: Figure S1. Phylogenetic analysis of H. marmoreus and the other 26 fungal species based on protein sequences of 14 conserved mitochondrial orthologous genes. A max likely hood species tree of 27 fungal species was constructed using RAxML and a bootstrap analysis with 1000 replications was performed. All of the bootstrap values at any node were 100%. The order, sub-kingdom, and kingdom corresponding to each species show their taxonomic classifications. [file 12864_2019_6133_MOESM1_ESM.jpg]

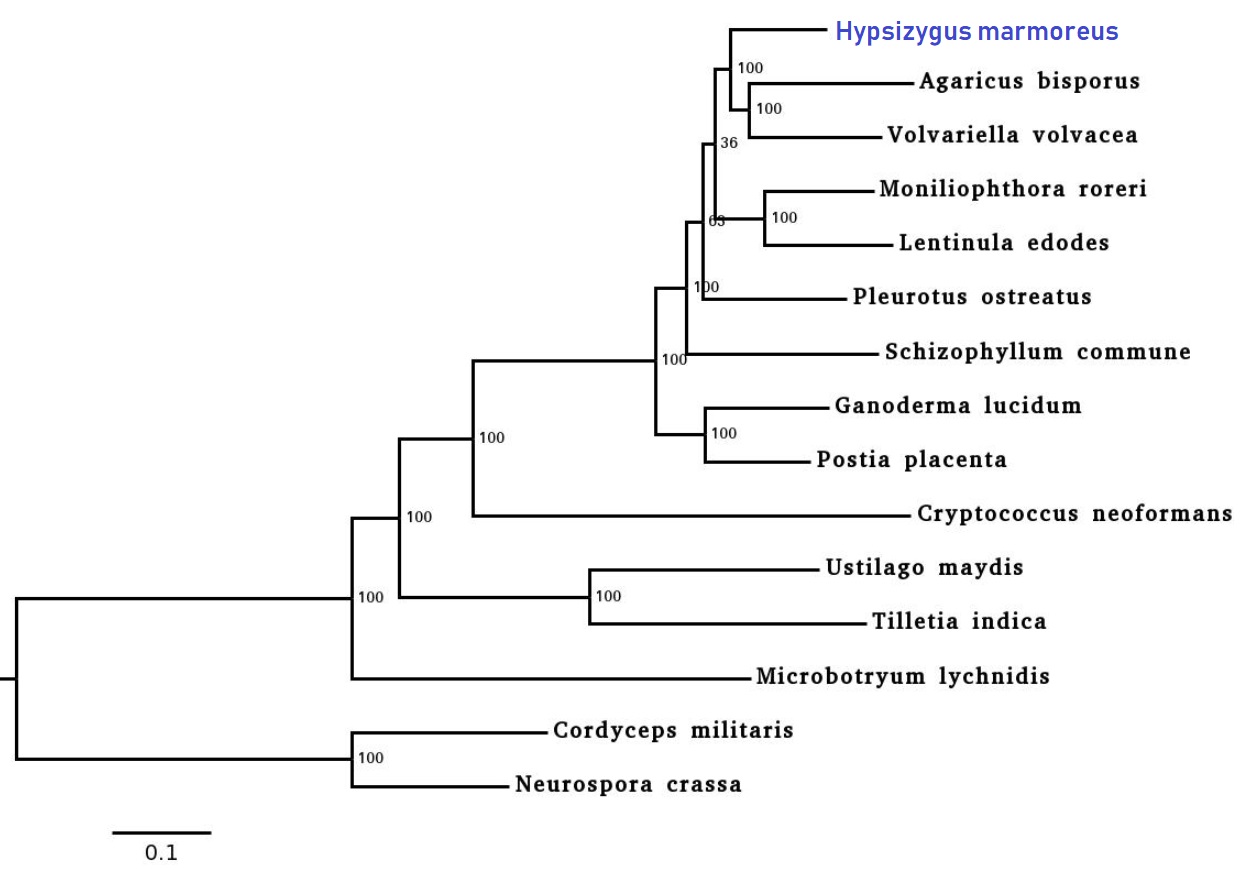

Supplement: Supplementary file 2 — Additional file 2: Figure S2. Phylogenetic analysis of H. marmoreus and 14 other fungal species based on a single copy of a homologous gene. A maximum likely hood species tree of 15 fungal species was constructed using RAxML and a bootstrap analysis with 1000 replications was performed. All of the bootstrap values at any node were 100%. [file 12864_2019_6133_MOESM2_ESM.jpg]

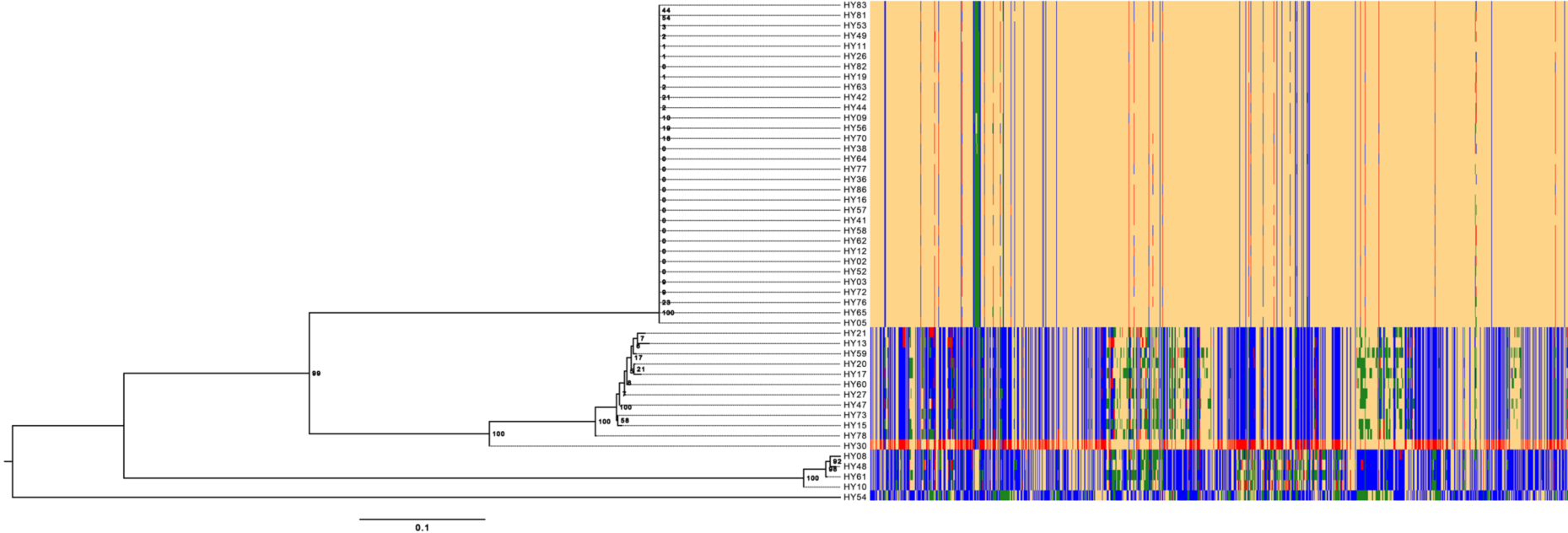

Supplement: Supplementary file 3 — Additional file 3: Figure S3. The phylogenetic and SNP analyze of H. marmoreus intraspecific. a. Phylogenetic tree construction of 48 HM strains using 972 mitochondrial genome SNP sites; b. Genotypes of 972 mitochondrial SNP loci in 48 HM strains, calculated as diploid. Yellow: the loci are homozygous and consistent with the reference genome; blue: loci are homozygous and inconsistent with the reference genome; red: loci are heterozygous; green: DNA sequencing reads are not aligned at this locus result. [file 12864_2019_6133_MOESM3_ESM.jpg]

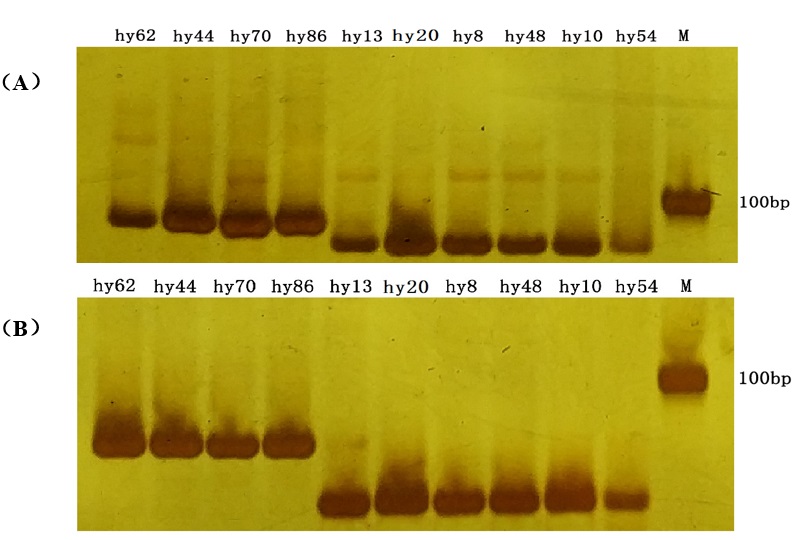

Supplement: Supplementary file 4 — Additional file 4: Figure S4. PAGE diagram of PCR products for two InDdels markers in different H. marmoreus strains. (A) InDel (GGGGTCCCGTAC/G) located at position 93,608 on the mt genomes; (B) InDel (TAGTAA/T) located at position 93,344 on themt genomes. The strains HM44, HM70 and HM86 were the same as HM62 (references genome) in group I, while, strains HM13, HM8, HM48, HM10 and HM54 were classified in group II, and III. M = marker. [file 12864_2019_6133_MOESM4_ESM.jpg]

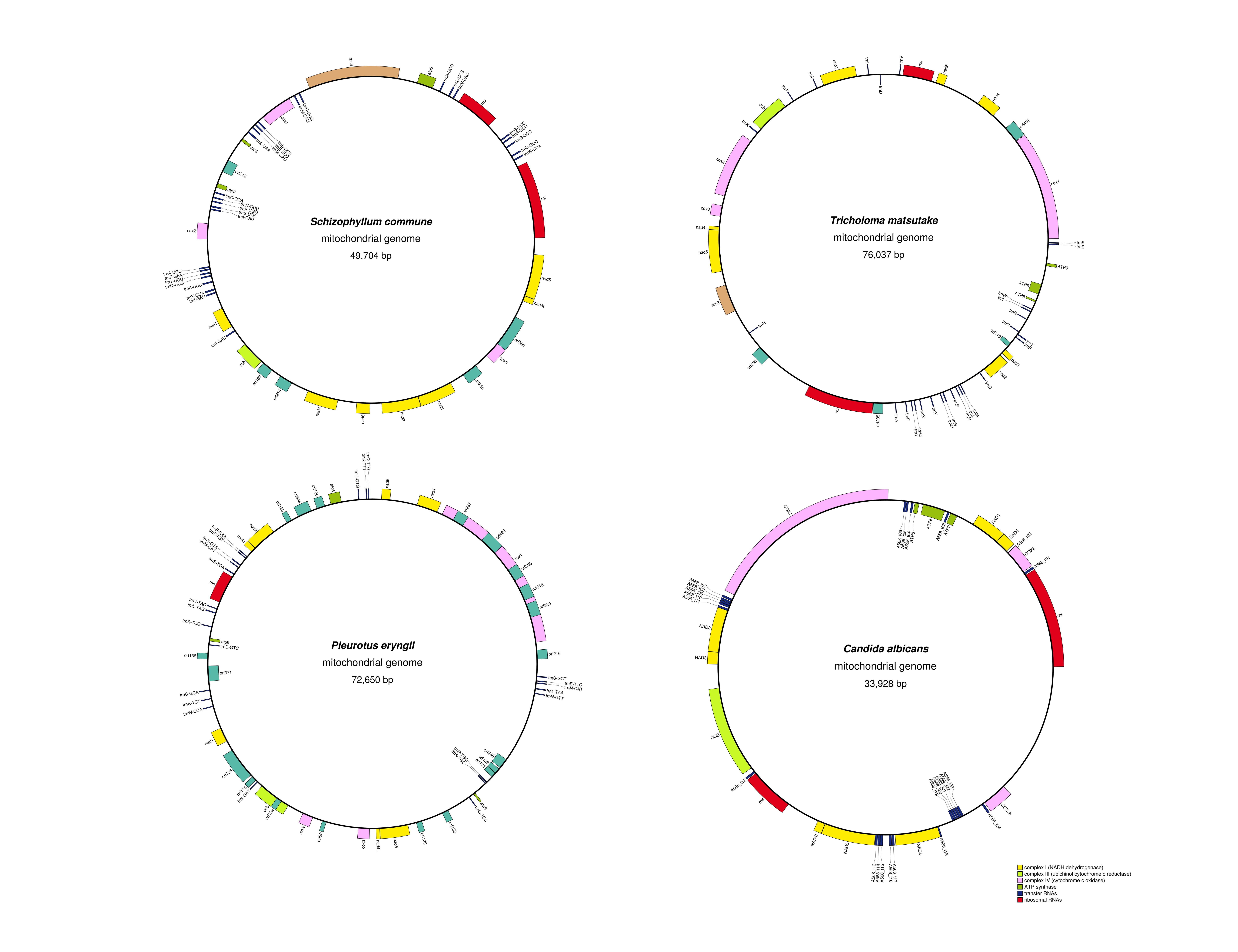

Supplement: Supplementary file 5 — Additional file 5: Figure S5. Circle diagrams representing the mitochondrial genomes of 4 different strains. [file 12864_2019_6133_MOESM5_ESM.jpg]

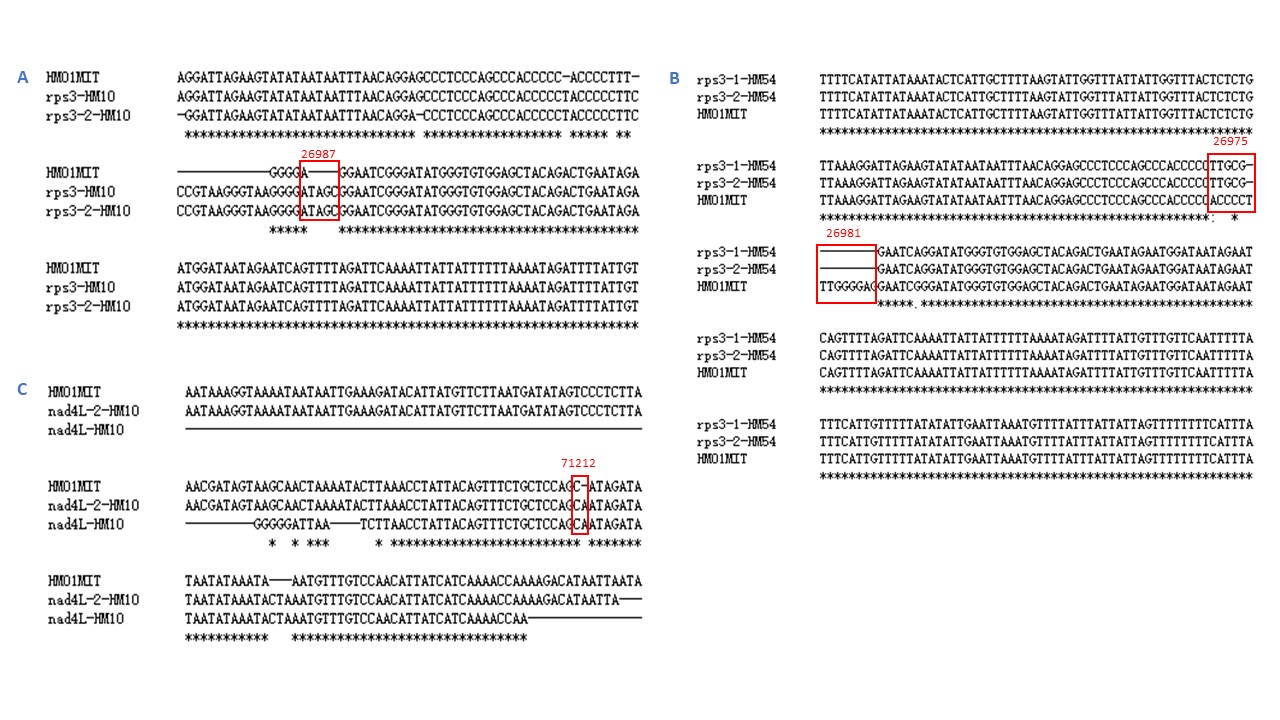

Supplement: Supplementary file 6 — Additional file 6: Figure S6. PCR product sequencing of the variant sites between HM62 and HM10 strains. (A) At 26987 location on the mt genome, there is a 4 bp InDels in rps3 of HM10. Rps3-2-HM10 and rps3-HM10 are two primers of PCR products. (B) At position 26,975 on the mt genome there is a 4 bp continuous point mutation (ACCCC/TTCGC) and 9 bp deletion (TTTGGGGAG) in rps3 of HM54. rps3-HM54 and Rps3-2-HM54 are two primers of PCR products. (C) At position 71,212 on the mt genome, there is a 2 bp InDels (C/CA) in nad4L of HM10. Nad4L-10 and Nad4L-2-10 are two primers of PCR products. [file 12864_2019_6133_MOESM6_ESM.jpg]
