# Supplementary material for: Mitochondrial genome in Hypsizygus marmoreus and its evolution in Dikarya
Source: BMC Genomics. 2019 Oct 22;20:765. doi: 10.1186/s12864-019-6133-z (PMC6805638; doi:10.1186/s12864-019-6133-z)
Supplement: Supplementary file 7 — Additional file 7: Table S1. Source of the 48 H. marmoreus strains. [file 12864_2019_6133_MOESM7_ESM.doc]

**Table S1. Source of the 48 *Hypsizigus marmoreus* strains**

| **Strain** | **Initial source of the strains** | **Second source of the strains** |
| --- | --- | --- |
| HM02 | Edible Fungus Room of Fuzhou Agricultural Science Research Institute | Mycological Research Center of Fujian Agriculture and Forestry University |
| HM03 | Donated by ShangGuan | Mycological Research Center of Fujian Agriculture and Forestry University |
| HM05 | Longhai Jiuhu Edible Fungus Research Institute of Fujian Province | Mycological Research Center of Fujian Agriculture and Forestry University |
| HM08 | Donated by Shang guan | Mycological Research Center of Fujian Agriculture and Forestry University |
| HM09 | Donated by Xiaoyu Li | Mycological Research Center of Fujian Agriculture and Forestry University |
| HM10 | Donated by Xiaoyu Li | Mycological Research Center of Fujian Agriculture and Forestry University |
| HM11 | Northeast Edible Medicinal Fungi Institute | Mycological Research Center of Fujian Agriculture and Forestry University |
| HM12 | Northeast Edible Medicinal Fungi Institute | Mycological Research Center of Fujian Agriculture and Forestry University |
| HM13 | Jiangdu Tianda Edible Fungus Research Institute | Mycological Research Center of Fujian Agriculture and Forestry University |
| HM15 | Strain Research Center of Huazhong Agricultural University | Mycological Research Center of Fujian Agriculture and Forestry University |
| HM16 | Shouguang Edible Fungi Institute of Shandong Province | Mycological Research Center of Fujian Agriculture and Forestry University |
| HM17 | Sanming Mycological Research Institute of Fujian Province | Mycological Research Center of Fujian Agriculture and Forestry University |
| HM19 | Strain Research Center of Huazhong Agricultural University | Mycological Research Center of Fujian Agriculture and Forestry University |
| HM20 | Longhai Jiuhu Edible Fungus Reearch Institute of Fujian Province | Mycological Research Center of Fujian Agriculture and Forestry University |
| HM21 | Xijiu Edible Fungi Research Center | Mycological Research Center of Fujian Agriculture and Forestry University |
| HM26 | Mycological Research Institute of Miyang County, Henan Province | Mycological Research Center of Fujian Agriculture and Forestry University |
| HM27 | Huanyu Edible Fungus Research Institute of Jiayu Country,Hubei Province | Mycological Research Center of Fujian Agriculture and Forestry University |
| HM36 | Longhai Jiuhu Edible Fungus Research Institute of Fujian Province | Mycological Research Center of Fujian Agriculture and Forestry University |
| HM38 | Institute of Plant Protection of Fujian Academy of Agricultural Sciences | Mycological Research Center of Fujian Agriculture and Forestry University |
| HM41 | Yatai Edible Fungus Research Institute of Jinzhou, liaoning province | Mycological Research Center of Fujian Agriculture and Forestry University |
| HM42 | Edible Fungus Research Institute of Hunan Agricultural University | Mycological Research Center of Fujian Agriculture and Forestry University |
| HM44 | Mycological Research Center of Fujian Agriculture and Forestry University | N/A |
| HM47 | Mycological Research Center of Fujian Agriculture and Forestry University | N/A |
| HM48 | Mycological Research Center of Fujian Agriculture and Forestry University | N/A |
| HM49 | Mycological Research Center of Fujian Agriculture and Forestry University | N/A |
| HM52 | Mycological Research Center of Fujian Agriculture and Forestry University | N/A |
| HM53 | Edible Fungus Research Institute of Heilongjiang Province | N/A |
| HM54 | Edible Fungus Research Institute of Heilongjiang Province | N/A |
| HM56 | Tianda Edible Fungus Research Institute of Jiangsu Province | N/A |
| HM57 | The Mudanjiang Branch of Heilongjiang Academy of Agricultural Sciences | Center for Genomics and Biotechnology of Fujian Agriculture and Forestry University |
| HM58 | The Mudanjiang Branch of Heilongjiang Academy of Agricultural Sciences | N/A |
| HM59 | The Mudanjiang Branch of Heilongjiang Academy of Agricultural Sciences | Center for Genomics and Biotechnology of Fujian Agriculture and Forestry University |
| HM60 | The Mudanjiang Branch of Heilongjiang Academy of Agricultural Sciences | Center for Genomics and Biotechnology of Fujian Agriculture and Forestry University |
| HM61 | Vanchen Mushrooms of Zhangzhou | N/A |
| HM62 | Fuquanxin Edible Fungus Limited Liability Company of Gutian County | N/A |
| HM63 | Japan | Center for Genomics and Biotechnology of Fujian Agriculture and Forestry University |
| HM64 | USYD ( University of Sydney ) | Center for Genomics and Biotechnology of Fujian Agriculture and Forestry University |
| HM65 | Chinese Academy of Agricultural Sciences | Center for Genomics and Biotechnology of Fujian Agriculture and Forestry University |
| HM70 | Chinese Academy of Agricultural Sciences | Agricultural Culture Collection of China |
| HM72 | Microbiological Culture Collection Center of Sandong Agricultural University | Center for Genomics and Biotechnology of Fujian Agriculture and Forestry University |
| HM73 | Guangdong Institute of Microbiology | Center for Genomics and Biotechnology of Fujian Agriculture and Forestry University |
| HM76 | Research Institute of Subtropical Forestry,Chinese Academy of Forestry | Center for Genomics and Biotechnology of Fujian Agriculture and Forestry University |
| HM77 | Xiangshan of Japan | Center for Genomics and Biotechnology of Fujian Agriculture and Forestry University |
| HM78 | Sichuan Agricultural University | Center for Genomics and Biotechnology of Fujian Agriculture and Forestry University |
| HM81 | The Thousand-buddha Mountain of Mianyang,Sichuan Province | Center for Genomics and Biotechnology of Fujian Agriculture and Forestry University |
| HM82 | Weifang City, Shandong | Center for Genomics and Biotechnology of Fujian Agriculture and Forestry University |
| HM83 | Fujiyama of Japan | China Forestry Culture Collection Center |
| HM86 | Fujian Shunchang Shennong mushroom industry co. LTD | N/A |
