# Supplementary material for: Mitochondrial genome in Hypsizygus marmoreus and its evolution in Dikarya
Source: BMC Genomics. 2019 Oct 22;20:765. doi: 10.1186/s12864-019-6133-z (PMC6805638; doi:10.1186/s12864-019-6133-z)
Supplement: Supplementary file 8 — Additional file 8: Table S2. Statistics for the general features of mt genomes among 26 fungal species. [file 12864_2019_6133_MOESM8_ESM.doc]

**Table S2. Statistics for the general features of mt genomes among 26** fungal species

| **Species** | **Length** | **GC** | **Coding gene** | **tRNA** | **rRNA** | **Accession** |
| --- | --- | --- | --- | --- | --- | --- |
| *Phlebia radiata* | 156348 | 0.31 | 126 | 28 | 2 | NC_020148 |
| *Agaricus bisporus* | 135005 | 0.29 | 44 | 35 | 2 | JX271275 |
| *Lentinula edodes* | 121394 | 0.31 | 27 | 28 | 2 | NC_0183654 |
| *Crinipellis perniciosa* | 109103 | 0.31 | 14 | 26 | 2 | AY376688 |
| *Microbotryum lychnidis* | 107808 | 0.34 | 51 | 31 | 1 | NC_020353 |
| *Moniliophthora roreri* | 93722 | 0.28 | 56 | 26 | 2 | NC_015400 |
| *Trametes cingulata* | 91500 | 0.24 | 20 | 25 | 2 | NC_013933 |
| *Flammulina velutipes* | 88508 | 0.16 | 31 | 26 | 2 | NC_021373 |
| *Tricholoma matsutake* | 76037 | 0.21 | 24 | 28 | 2 | NC_028135 |
| *Pleurotus ostreatus* | 73242 | 0.26 | 26 | 24 | 2 | NC_009905 |
| *Pleurotus eryngii* | 72650 | 0.26 | 40 | 23 | 1 | NC_033533 |
| *Tilletia indica* | 65147 | 0.29 | 14 | 24 | 2 | NC_009880 |
| *Neurospora crassa* | 64840 | 0.36 | 28 | 28 | 0 | NC_026614 |
| *Piriformospora indica* | 63682 | 0.26 | 53 | 23 | 0 | FQ859090 |
| *Ganoderma lucidum* | 60635 | 0.27 | 22 | 27 | 2 | NC_021750 |
| *Tilletia walkeri* | 59352 | 0.29 | 14 | 24 | 0 | NC_010651 |
| *Cantharellus cibarius* | 58656 | 0.27 | 33 | 26 | 2 | NC_020368 |
| *Ustilago maydis* | 56814 | 0.31 | 26 | 23 | 2 | DQ157700 |
| *Schizophyllum commune* | 49704 | 0.22 | 20 | 27 | 2 | NC_003049 |
| *Rhodotorula taiwanensis* | 40392 | 0.41 | 25 | 23 | 2 | HF558455 |
| *Pichia pastoris* | 35683 | 0.22 | 16 | 27 | 2 | NC_015384 |
| *Candida albicans* | 33928 | 0.32 | 14 | 26 | 2 | NC_018046 |
| *Cordyceps militaris* | 33277 | 0.27 | 15 | 27 | 2 | NC_022834 |
| *Phakopsora meibomiae* | 32520 | 0.35 | 15 | 24 | 2 | NC_014352 |
| *Phakopsora pachyrhizi* | 31825 | 0.35 | 15 | 24 | 2 | NC_014344 |
| *Jaminaea angkorensis* | 29999 | 0.32 | 18 | 22 | 2 | NC_023248 |
