# Supplementary material for: Mitochondrial genome in Hypsizygus marmoreus and its evolution in Dikarya
Source: BMC Genomics. 2019 Oct 22;20:765. doi: 10.1186/s12864-019-6133-z (PMC6805638; doi:10.1186/s12864-019-6133-z)
Supplement: Supplementary file 9 — Additional file 9: Table S3. Variation site statistics for the mt genome of H. marmoreus. [file 12864_2019_6133_MOESM9_ESM.doc]

**Table S3. Variation site statistics for the mt genome of *H. marmoreus***

| **Method** | **# Variant** | **# SNP** | **# InDel** |
| --- | --- | --- | --- |
| GATK | 2249 | 1500 |  |
| Samtools | 2704 | 1467 |  |
| Intersection of GATK and Samtools | 1831 | 1307 |  |
| After hard filtering | 1373 | 972 |  |
