# Supplementary material for: Mitochondrial genome in Hypsizygus marmoreus and its evolution in Dikarya
Source: BMC Genomics. 2019 Oct 22;20:765. doi: 10.1186/s12864-019-6133-z (PMC6805638; doi:10.1186/s12864-019-6133-z)
Supplement: Supplementary file 10 — Additional file 10: Table S4. Statistics for the genotypes of 48 strains. [file 12864_2019_6133_MOESM10_ESM.doc]

Table S4. Statistics for the genotypes of 48 strains

| **Genotype** | **# Number** |
| --- | --- |
| Homozygosis and same to the reference site (0/0) | 37834 |
| Homozygosis and different to the reference site (1/1, 2/2 or 3/3) | 6954 |
| Not Detected (./.) | 1587 |
| Heterozygosis (0/1, 0/2, 0/3, 1/2, 1/3, 2/3) | 281 |
| Total | 46656 (972 * 48) |
