# Supplementary material for: Mitochondrial genome in Hypsizygus marmoreus and its evolution in Dikarya
Source: BMC Genomics. 2019 Oct 22;20:765. doi: 10.1186/s12864-019-6133-z (PMC6805638; doi:10.1186/s12864-019-6133-z)
Supplement: Supplementary file 12 — Additional file 12: Table S6. Effects of variants on 15 mt conserved genes. [file 12864_2019_6133_MOESM12_ESM.doc]

**Table S6. Effects of variants on 15 mt conserved genes**

| **Designation** | **Total** | **Ratio** | **atp6** | **atp8** | **atp9** | **cob** | **cox1** | **cox2** | **cox3** | **nad1** | **nad2** | **nad3** | **nad4** | **nad4L** | **nad5** | **nad6** | **rnl** | **rns** | **rps3** |
| --- | --- | --- | --- | --- | --- | --- | --- | --- | --- | --- | --- | --- | --- | --- | --- | --- | --- | --- | --- |
| frameshift_variant | 4 | 0.21% | 0 | 0 | 0 | 0 | 0 | 0 | 0 | 0 | 0 | 0 | 0 | 1 | 0 | 0 | 0 | 0 | 3 |
| stop_lost | 1 | 0.05% | 0 | 0 | 0 | 0 | 0 | 0 | 0 | 0 | 0 | 0 | 0 | 0 | 1 | 0 | 0 | 0 | 0 |
| conservative_inframe_deletion | 1 | 0.05% | 0 | 0 | 0 | 0 | 0 | 0 | 0 | 0 | 0 | 0 | 0 | 0 | 0 | 0 | 0 | 0 | 1 |
| conservative_inframe_insertion | 1 | 0.05% | 0 | 0 | 0 | 0 | 0 | 0 | 0 | 0 | 0 | 0 | 0 | 1 | 0 | 0 | 0 | 0 | 0 |
| missense_variant | 18 | 0.93% | 0 | 0 | 0 | 3 | 1 | 0 | 0 | 0 | 5 | 1 | 0 | 0 | 0 | 1 | 0 | 0 | 7 |
| synonymous_variant | 50 | 2.58% | 5 | 0 | 1 | 4 | 9 | 2 | 0 | 1 | 9 | 2 | 8 | 1 | 3 | 0 | 0 | 0 | 5 |
| non_coding_transcript_exon_variant | 116 | 5.98% | 0 | 0 | 0 | 0 | 0 | 0 | 0 | 0 | 0 | 0 | 0 | 0 | 0 | 0 | 115 | 1 | 0 |
| intron_variant | 139 | 7.16% | 0 | 0 | 0 | 16 | 71 | 20 | 0 | 0 | 0 | 0 | 0 | 0 | 32 | 0 | 0 | 0 | 0 |
| intergenic_region | 1,189 | 61.26% | - | - | - | - | - | - | - | - | - | - | - | - | - | - | - | - | - |
| upstream_gene_variant | 210 | 10.82% | 9 | 12 | 9 | 16 | 0 | 17 | 14 | 22 | 11 | 5 | 9 | 16 | 9 | 13 | 22 | 21 | 5 |
| downstream_gene_variant | 212 | 10.92% | 17 | 18 | 10 | 6 | 13 | 13 | 19 | 12 | 9 | 14 | 15 | 4 | 7 | 15 | 14 | 14 | 12 |
