# Supplementary material for: Mitochondrial genome in Hypsizygus marmoreus and its evolution in Dikarya
Source: BMC Genomics. 2019 Oct 22;20:765. doi: 10.1186/s12864-019-6133-z (PMC6805638; doi:10.1186/s12864-019-6133-z)
Supplement: Supplementary file 13 — Additional file 13: Table S7. Primers used for H. marmoreus DNA amplification and sequencing. [file 12864_2019_6133_MOESM13_ESM.doc]

**Table S7. Primers used for *H. marmoreus* DNA ampliﬁcation and sequencing**

| **Primer** | **Gene or region ampliﬁed** | **Sequence 5’ > 3’** | **Amplicon size(bp)** |
| --- | --- | --- | --- |
| nad4L-L-HM10 | Nad4L | ATATAGTCCCTCTTAAACGAT | 100 |
| nad4L-R-HM10 | Nad4L | TGGTTTTGATGATAATGTTGG | 100 |
| nad4L-2-L-HM10 | Nad4L | AACAGGGGAACCACAGATACC | 311 |
| nad4L-2-R-HM10 | Nad4L | AATTATGTCTTTTGGTTTTGA | 311 |
| rps3-L-HM10 | Rps3 | AGAATAGAAAATAAATACACC | 366 |
| rps3-R-HM10 | Rps3 | GGGAAGGGATGGGAAGGGAGA | 366 |
| rps3-2-L-HM10 | Rps3 | ACTCATTGCTTTTAAGTATTG | 218 |
| rps3-2-R-HM10 | Rps3 | TTGAACAAACAATAAAATCTA | 218 |
| rps3-L-HM54 | Rps3 | TAGTCCCGGACAGGGTAGTT | 456 |
| rps3-R-HM54 | Rps3 | CGCAATCCCAGGCTTTACAC | 456 |
| rps3-2-L-HM54 | Rps3 | TTAGTCCCGGACAGGGTAGT | 458 |
| rps3-2-R-HM54 | Rps3 | CCGCAATCCCAGGCTTTACA | 458 |
| hy93608-L | 93608-L | TTTAATCTTGACCACCGCTTCG | 94 |
| hy93608-R | 93608-R | AAGGAGAAGATCAGGAGTAGTC | 94 |
| hy93344-L | 93344-L | ACCGAGACTTCTTTCCCCAATC | 87 |
| hy93344-R | 93344-R | GGTCTCACCCCTGCCCCTTATC | 87 |
