# Supplementary material for: Mitochondrial genome in Hypsizygus marmoreus and its evolution in Dikarya
Source: BMC Genomics. 2019 Oct 22;20:765. doi: 10.1186/s12864-019-6133-z (PMC6805638; doi:10.1186/s12864-019-6133-z)
Supplement: Supplementary file 14 — Additional file 14: Table S8. The distances between nad2, nad3 and between nad4L, nad5. [file 12864_2019_6133_MOESM14_ESM.doc]

**Table S8. The distance of gene pairs of *nad2, nad3 and nad4L, nad5***

| **Species** | **Distance of nad2/nad3** | **Distance of nad4L/nad5** |
| --- | --- | --- |
| *Hypsizigus marmoreus* | -1 | 61 |
| *Tricholoma_matsutake* | 184 | -1 |
| *agaricus_bisporus* | -1 | 0 |
| *Pleurotus_ostreatus* | 512 | 674 |
| *Pleurotus_eryngii* | 3 | -1 |
| *Crinipellis_perniciosa* | -1 | 1084 |
| *Moniliophthora_roreri* | -1 | 1 |
| *Lentinula_edodes* | -1 | 2 |
| *Flammulina_velutipes* | 0 | 3 |
| *Schizophyllum_commune* | 0 | 2 |
| *Trametes_cingulata* | 1 | 1365 |
| *Ganoderma_lucidum* | 1 | -1 |
| *Phlebia_radiata* | 5331 | 0 |
| *Piriformospora_indica* | 390 | 29 |
| *Cantharellus_cibarius* | 2 | -1 |
| *Phakopsora_pachyrhizi* | 1 | 2 |
| *Phakopsora_meibomiae* | 1 | 2 |
| *Microbotryum_lychnidis* | -1 | 0 |
| *Rhodotorula_taiwanensis* | 5 | 0 |
| *Ustilago_maydis* | -1 | -1 |
| *Jaminaea_angkorensis* | -1 | -1 |
| *Tilletia_indica* | 512 | 674 |
| *Tilletia_walkeri* | 512 | 672 |
| *Candida_albicans* | -1 | -1 |
| *Pichia_pastoris* | -1 | -1 |
| *Neurospora_crassa* | 1527 | 403 |
| *Cordyceps_militaris* | -1 | -1 |

Note: The orientation of nad2/nad3 and nad4L/nad5 on the mitochondrial genome is consistent.

(a). 0 indicates a difference of 0 bases between the two genes, the stop codon of the previous gene and the start codon of the latter gene; (b). -1 indicates that there is a base overlap of 1bp between the stop codon of the previous gene and the start codon of the latter gene; (c). A value greater than 0 indicates the base distance between the stop codon of the previous gene and the start codon of the latter gene by a specified number.
